# Spectacle Coverage and Spectacles Use among Elderly Population in Residential Care in the South Indian State of Andhra Pradesh

**DOI:** 10.1155/2013/183502

**Published:** 2013-06-23

**Authors:** Srinivas Marmamula, L. V. Chandra Sekhar Ravuri, Mei Ying Boon, Rohit C. Khanna

**Affiliations:** ^1^Allen Foster Community Eye Health Research Centre, International Centre for Advancement of Rural Eye care, L V Prasad Eye Institute, L V Prasad Marg, Banjara Hills, Hyderabad 500 034, India; ^2^Bausch & Lomb School of Optometry, L V Prasad Eye Institute, Hyderabad 500 034, India; ^3^Dana Center for Preventive Ophthalmology, Wilmer Eye Institute at Johns Hopkins University, Baltimore, MD 21287, USA; ^4^School of Optometry & Vision Science, University of New South Wales, Sydney 2033, Australia; ^5^Community Health & Nutrition Office, Area Hospital, Kandukur, Prakasam District, Andhra Pradesh 523105, India

## Abstract

*Background*. There is limited research conducted on uncorrected refractive errors, presbyopia, and spectacles use among the elderly population in residential care in developing countries such as India. We conducted a cross-sectional study among elderly in residential care to assess the spectacle coverage and spectacles usage in the south Indian state of Andhra Pradesh. *Methods*. All 524 residents in the 26 “homes for aged” institutions in the district were enumerated. Eye examination was performed that included visual acuity (VA) assessment for distant and near vision. A questionnaire was used to collect information on spectacles use. *Results*. 494/524 individuals were examined, 78% were women, and 72% had no education. The mean age of participants was 70 years. The spectacle coverage for refractive errors was 35.1% and 23.9% for presbyopia. The prevalence of current use and past use of spectacles was 38.5% (95% CI: 34.2–42.8; *n* = 190) and 17.2% (95% CI: 13.9–42.8), respectively. *Conclusions*. There is low spectacle coverage for both refractive errors and presbyopia among elderly individuals in residential care in the south Indian state of Andhra Pradesh. Appropriate service delivery systems should be developed to reach out this vulnerable group of seniors on a priority basis.

## 1. Introduction 

Uncorrected refractive errors remain the leading cause of visual impairment worldwide [1]. It is one of the priorities of VISION 2020, The Right to Sight initiative. Uncorrected refractive errors that result in visual impairment are termed as correctable visual impairment by few authors [2]. Presbyopia is now recognized as a major problem affecting over a billion people globally out of which 517 million are in need of correction for near vision [3]. By far, spectacles remain the most common and cost-effective intervention for refraction errors and presbyopia worldwide. Despite this, the data on spectacle usage patterns is limited. 

There is limited research conducted on uncorrected refractive errors and spectacles use among the elderly population in residential care in developing countries such as India. Elderly people living in residential care are a vulnerable group and tend to have a higher prevalence of visual impairment compared to the general population as evidenced in studies from developed countries [4–9]. Higher prevalences were also found in the studies conducted in developing countries [10–12]. In both developed and developing countries, uncorrected refractive errors were major causes of visual impairment. 

In a recent study among the elderly in residential care in the state of Andhra Pradesh, we discovered that uncorrected refractive errors were the second leading cause of visual impairment after cataract [13]. This follow-up paper from the same study extends beyond prevalence and causes of visual impairment and reports on uncorrected refractive errors, spectacles use and coverage. We also report on uncorrected presbyopia among this elderly population. As both refractive errors and presbyopia can be easily addressed through primary eye care approach of eye screening by paramedical personnel and dispensing of spectacles, our results have implications for planning such services for elderly population in residential care in India. The data from both papers together provide a comprehensive overview of the eye care needed among elderly population in residential care in the south Indian state of Andhra Pradesh.

## 2. Methods

The study was approved by the institutional review board of L V Prasad Eye Institute, Hyderabad, Indian and followed the tenets of declaration of Helsinki. Written informed consent was obtained from all the study participants before commencing with the study procedures.

The details of the eye examination protocol are described elsewhere [13]. In brief, a team consisting of an ophthalmic officer (paramedical ophthalmic personnel) and a field assistant visited the “home for the aged” institutions and conducted eye examinations. Distant and near visual acuity (VA) were assessed. A questionnaire was used to collect information on present and past spectacles use, the spectacles provider, and the purpose of using the spectacles. The spectacles providers were classified as private eye clinics (small clinics usually staffed by paramedical ophthalmic personnel who are available and eye examination is performed; few clinics also have a visiting ophthalmologist at a regular frequency), nongovernment eye hospitals, optical shops (shops where there are no formally trained eye care personnel available and spectacles are prescribed based on subjective correction or based autorefraction), and eye camps (make-shift eye clinics where refraction is performed and spectacles are provided free of cost).

To calculate the spectacle coverage, “met need” was defined as the number of subjects who were using their spectacles to correct refractive error and/or presbyopia. For refractive errors, all subjects who had unaided VA < 6/18 in either eye, which improved to 6/18 or better with spectacles, were considered to have had their need met; that is, their refractive error was corrected. “Unmet need” was defined as the number of subjects who had unaided VA < 6/18 in either eye but improved to 6/18 or better with pinhole and had no spectacles. Total need, which is the sum of met and unmet needs, gives the prevalence of refractive error in the population. The concept of spectacle coverage is similar to that used in previous studies [14, 15]. The spectacle coverage (%) for refractive errors was calculated as follows: spectacle coverage (%) = (met need/met need + unmet need) × 100. 

For presbyopia, “met need” was defined as unaided near vision <N8, but improved to N8 or better with the spectacles they were using. “Unmet need” was defined as unaided near vision <N8 and had no spectacles for near vision but improved to N8 or better with a near addition, that is, uncorrected presbyopia. Total need, which is the total of unmet and met needs, gives the prevalence of presbyopia in the population. The same formula described earlier was used to calculate the spectacle coverage for presbyopia. The N8 criterion was used as a conservative estimate of corrected presbyopia. It also corresponds to newsprint in India, and this criterion was also used in previous studies [14, 16].

Data management and analysis were done using SPSS 16.0 (SPSS Inc., Chicago, IL, USA). Point prevalence estimates and 95% CI (confidence intervals) were calculated. Multiple logistic regression was used to assess the association between current use of spectacles and demographic variables. Adjusted odds ratio (OR) with 95% CI is presented.

## 3. Results

### 3.1. Characteristics of the Study Sample

A total of 524 subjects were enumerated from 26 institutions for the elderly, and 494 (94.3%) were examined. Data was not available on 15 participants who had systemic conditions and hence eye examination was not possible. Another 15 subjects were not available during the visit. Among those who were examined, 78.1% of the subjects were female, and 72.1% had no education. Nearly 57% of those examined were aged 70 years or older. The mean age of the participants was 70 years (standard deviation = 8.6 years). 

### 3.2. Refractive Errors, Presbyopia, and Spectacle Coverage

Refractive errors were present in 114/494 (23.1%; 95% CI: 19.4–26.8) individuals. These were uncorrected (unmet need) in 74 individuals (7.8%; 95% CI: 5.4–10.2) and corrected (met need) in 40 individuals. Based on these, the spectacle coverage for refractive errors was 35.1%. The spectacle coverage for refractive errors was higher among older individuals, men, and those with any education. The unmet need for refractive errors was associated significantly with the level of education (*P* < 0.01); no significant association was found with age and gender (Table 1).

Presbyopia was present in 271 individuals (55.1%; 95% CI: 50.7–59.4). It was uncorrected (unmet need) in 207 individuals (41.9%; 95% CI: 73.5–46.3) and corrected (met need) in 65 individuals. Based on this the spectacle coverage for presbyopia was 23.9%. The spectacle coverage for presbyopia was higher among men and those with any education. The unmet need for presbyopia was significantly associated with age (*P* < 0.01) and levels of education but not with gender (Table 1). 

### 3.3. Spectacles Use

The prevalence of current spectacles use was 38.5% (95% CI: 34.2–42.8; *n* = 190). It was significantly higher in older individuals (*P* < 0.01) and those with education (*P* < 0.01). Though spectacle use was higher among men than women, it was not statistically significant (*P* = 0.15). A total of 85/494 (17.2%; 95% CI: 13.9–42.8) individuals reported the use of spectacles in the past. The common reasons quoted for discontinuation of spectacles were “lost spectacles and no money to buy a new pair” (*n* = 40/85; 44.7%), “no benefit from use of spectacles” (*n* = 23/85; 27.1%), “broken or scratches on the lenses” (*n* = 19/85; 24.8%), and “discomfort with spectacles” (*n* = 3/85; 3.5%). The past use of spectacles was not associated with age, gender, or education (Table 2).

On applying multiple logistic regression, the current use of spectacles was associated with older age, any level of education and history of surgery in either eye. Though the odds of spectacles use were higher among women than men, it was not statistically significant (Table 3).

Among the current users of spectacles, the single vision for distance was the most commonly used type of spectacles (*n* = 97; 51.1%) followed by bifocals (*n* = 86; 45.3%). Single vision spectacles for near vision only were being used by seven individuals (3.7%). Private eye clinics (*n* = 138; 72.6%) were the leading service providers for spectacles followed by local nongovernment organizations (*n* = 25; 13.2). Fourteen individuals (7.4%) procured their spectacles directly from an optical shop, and the remaining 13 (6.8%) individuals got their spectacles at no cost from a government screening camp. 

## 4. Discussion

We found a high prevalence of uncorrected refractive errors, presbyopia, and low spectacle coverage in elderly population in residential care in the south Indian state of Andhra Pradesh. Previously, we had reported a high prevalence of visual impairment in this population out of which over 26% was due to uncorrected refractive errors [13]. Several papers have reported the prevalence of uncorrected refractive errors in the elderly in general population [2, 11, 17–19], 10% in Taiwan [11], 3.0% and 3.2% in the United Kingdom [8, 17, 20], 13.4% in Hong Kong [12] compared to 7.8% in the present study. The cross-comparisons of the results across the studies are of limited value owing to differences in the definition of uncorrected refractive errors, study settings, and population. The data from population-based cross-sectional studies cannot be used to compare our findings from residential care as both populations are very different. The residential care institutions tend to have more elderly people who are predominantly women.

Uncorrected refractive errors are the easiest of the public eye heath challenges that can be addressed, especially in elderly people in residential care. It is cost effective and the benefit is immediate which in turn can vastly improve the quality of life of these people [21].

We found a prevalence of presbyopia that was similar to a previous study from the state of Andhra Pradesh [16]. However our reported prevalence was higher than that reported from a previous study that had a younger age group [14], which was expected. Both of these studies were population-based compared to a specific group of elderly population in the present study. 

Spectacles use was not frequently reported in studies on the elderly. The study from Andhra Pradesh reported 17.4% prevalence of current spectacles use [22, 23], and the Chennai study revealed a prevalence of 17.6% and 52.6% in the rural and urban samples, respectively [23]. While the study from Andhra Pradesh included participants of all ages, the Chennai study was limited to those aged ≥40 years [22, 23]. Our finding of prevalence of 38.5% was higher than that reported from Andhra Pradesh and rural segment of Chennai study. However, it is less than that found in urban area in the Chennai study. The spectacle coverage for refractive errors was previously reported as 28.4% and 29% compared to 35% in the present study [14, 15]. Similarly the spectacle coverage for presbyopia was reported as 11.1% and 19% compared to 24% in the present study [14, 15]. The higher coverage can be attributed to the older population in the present study compared to the other two studies. 

The use of pinhole based definition for refractive errors as a surrogate measure of refractive errors, though helpful for rapid evaluation of the individuals who could benefit from spectacle correction, is not free from limitations. The results may vary if refraction was conducted on all the individuals. The inclusion of data regarding quality of life and visual function of participants could have provided more insights; however, these were not assessed due to time constraints. The visual needs of older people in residential care may differ from those in the general population and other populations, so this may be an important area for future research. 

In summary, we found a high prevalence of uncorrected refractive errors, uncorrected presbyopia, and low spectacle coverage. Appropriate service delivery systems including eye screening and spectacles dispensing programmes should be developed to reach out this vulnerable group of seniors on a priority basis. The correction of refractive errors and presbyopia may indirectly impact their general well-being, quality of life and contribute to a more independent living. 

## Figures and Tables

**Table 1:** Spectacle coverage for refractive errors and presbyopia.

	Total in the group	Refractive errors			Presbyopia		
	*n*	Met need *n* (%)	Unmet need *n* (%)^†^	Spectacle coverage (%)	Met need *n* (%)	Unmet need *n* (%)^‡^	Spectacle coverage (%)
Age group (years)							
50–69	214	11 (5.1)	35 (16.4)	23.9	26 (12.1)	77 (36.0)	25.2
70 and above	280	29 (10.4)	39 (13.9)	42.6	39 (13.9)	130 (46.4)	23.1
Gender							
Male	108	9 (8.3)	12 (11.1)	42.9	26 (24.1)	43 (39.8)	37.7
Female	386	31 (8.0)	62 (16.1)	33.3	39 (10.1)	164 (42.5)	19.2
Education level							
No education	356	17 (4.8)	65 (18.3)	5.3	19 (5.3)	168 (47.2)	10.2
Any education	138	23 (16.7)	9 (6.5)	33.3	46 (33.3)	39 (28.3)	54.1
Total	**494**	**40 (8.1)**	**74 (15.0)**	**35.1**	**65 (13.2)**	**207 (41.9)**	**23.9**

^†^Not statistically significant for age group and gender; *P* < 0.01 for education.

^‡^ *P* < 0.01 for age and gender; not statistically significant for gender.

Met need (refractive errors): defined as unaided visual acuity <6/18 in either eye, which improved to 6/18 or better with spectacles.

Unmet need (refractive errors): defined as the number of subjects who had unaided visual acuity <6/18 in either eye but improved to 6/18 or better with pinhole and had no spectacles.

Met need (presbyopia): defined as unaided near vision <N8, but improved to N8 or better with the spectacles they were using.

Unmet need (presbyopia): defined as unaided near vision <N8 and had no spectacles, but improved to N8 or better with a near addition.

Spectacle coverage (refractive errors and presbyopia) (%) = met need/(met need + unmet need) × 100.

**Table 2:** Present and past spectacles uses.

	Total sample	Spectacles use—present^†^		Spectacles use—past^‡^	
	*n*	*n*	%	*n*	%
Age group (years)					
50–69	214	66	30.8	44	20.6
70 and above	280	124	44.3	41	14.6
Gender					
Male	108	48	44.4	17	15.7
Female	386	142	36.8	68	17.6
Education level					
No education	356	109	30.6	63	17.7
Any education	138	81	58.7	22	15.9
Total	**494**	**190**	**38.5**	**85**	**17.2**

^†^ *P* < 0.01 for present spectacles use in age groups and association with level of education; *P* = 0.15 for spectacles use and association with gender; ^‡^not statistically significant across age groups, gender, or education among past spectacles users.

**Table 3:** Multivariate analysis showing associations with spectacles use.

	Adjusted odds ratio	95% confidence interval	*P* value
Age group (years)			
50–69	1.0		
70 and above	1.7	1.1–2.6	0.00
Gender			
Male	1.0		
Female	1.3	0.8–2.2	0.28
Education level			
No education	1.0		
Any education	5.4	3.2–8.6	0.00
Surgery status			
Never operated	1.0		
Operated in either eye	3.7	2.4–5.6	0.02
